# Current Distribution in the Discharge Unit of a 10-Cell Vanadium Redox Flow Battery: Comparison of the Computational Model with Experiment

**DOI:** 10.3390/membranes12111167

**Published:** 2022-11-21

**Authors:** Artem Glazkov, Roman Pichugov, Pavel Loktionov, Dmitry Konev, Dmitry Tolstel, Mikhail Petrov, Anatoly Antipov, Mikhail A. Vorotyntsev

**Affiliations:** 1 EMCPS Department, Mendeleev University of Chemical Technology of Russia, 125047 Moscow, Russia; 2 Frumkin Institute of Physical Chemistry and Electrochemistry, Russian Academy of Sciences, 119071 Moscow, Russia; 3 Institute of Problems of Chemical Physics, Russian Academy of Sciences, 142432 Chernogolovka, Russia

**Keywords:** redox flow batteries, all-vanadium redox flow battery, membrane-electrode assembly, shunting currents, vanadium stack, destructive effect, electrolyte supply channel, capacity fade, current distribution

## Abstract

Shunting currents are among the main problems of all-vanadium redox flow battery stacks since, in addition to capacity losses, they cause negative effects associated with the local destruction of electrodes and bipolar plates. The values of both the shunting currents and their destructive effects on materials can be reduced at the battery development stage by adjusting the resistance of the electrolyte supply channels. The solution to this problem can be found using a calculation model for current distribution based on the current balance in the nodes as well as voltage drops and electromotive force in internal circuits according to Kirchhoff’s laws. This paper presents the verification of the model of current distribution in an all-vanadium redox flow battery stack of an original design that allows for the determination of membrane-electrode assembly resistances and electrolyte supply channels via direct measurements. Based on a comparison of the calculated and experimental values of the coulombic efficiency of charge–discharge cycles, the capacity fade associated with the crossover of vanadium compounds through the membrane has been determined.

## 1. Introduction

The growing share of alternative energy sources in global energy production has increased the demand for the development of energy storage systems. Solar, wind, and tide energy are characterized by an irregular nature in terms of electricity production. Most renewable energy sources are intermittent, which opens spatial and temporal gaps between the availability of energy and its consumption by the end-users. Hence, it is difficult to apply these valuable generated electric energies continuously and stably [1]. For their effective operation, various energy storage systems are required, which can shift the energy from off-peak demands to the peak demand period [2], thereby creating opportunities to limit the price of energy during the day.

Among electrochemical applications, redox flow batteries (RFB) occupy a special place in solving problems regarding the accumulation, storage, and secondary production of electricity. Structurally, they are similar to fuel cells consisting of a separated energy reservoir and reactor. The operation principle of RFB is close to that of a rechargeable battery, as both reversibly convert electrical energy into chemical energy. As a result, RFBs provide independent scaling of the energy storage volume and discharge power while acting as a secondary energy source [3,4].

The vanadium RFB (VRFB) is one of the most studied types. It demonstrates a good balance between key characteristics and up to 20,000 cycles of stable operation [5]. Megawatt-scale VRFBs are already used in the energy sectors of Japan, Australia, China, and other countries [6], while a number of companies are engaged in the manufacture and installation of container-type VRFBs for less energy-demanding applications. However, the wide distribution of VRFBs with respect to the current level of technical characteristics (power density, discharge current density, and stored energy density) is hindered by a relatively high vanadium electrolyte price, contributing half of the VRFB capital cost [7,8,9,10].

The research on vanadium redox flow batteries stems from single membrane-electrode assemblies. In the last decade, substantial efforts have been made to improve the key components and operating conditions of VRFB prototypes. Therefore, research on improving their performance characteristics is particularly topical within the following primary areas: the modification of ion-exchange membranes [11,12] and electrode materials [13,14,15], the optimization of electrolyte compositions [16,17,18], and flow field design [19,20]. The progress in these areas resulted in breakthroughs in the improvement of VRFB performance. The latest results show that a single membrane-electrode assembly (MEA) of a VRFB emits 2.78 W cm^−2^ and provides stable cycling for 20,000 cycles at 600 mA cm^−2^ with 81% energy efficiency and 78% electrolyte utilization [21].

The aforementioned results were obtained for cells with a single membrane-electrode assembly operating in the voltage range of 1.0–1.65 V. However, for practical applications, it is necessary to create a stack of MEAs because only an in series electrical connection of multiple cells allows for the attainment of the required level of operating power without a proportional increase in operating current followed by a proportional increase in ohmic loss. Once the operating voltage reaches a sufficient level, the ohmic energy losses during transmission and current conversion will be minimized. A number of other engineering aspects are central for a stack’s design. The main factors to be considered in multi-stack design are flow distribution and shunt current losses [2].

Shunting currents (SC) constitute a serious problem for VRFB stacks since, in addition to capacity losses, they cause negative effects associated with the local destruction of electrodes and bipolar plates. Reducing the values of both shunting currents and their destructive effect on materials is possible at the battery development stage by adjusting the resistance of the electrolyte supply channels that feed the electrode spaces by changing their geometry (length and cross-sectional area). By modelling this phenomenon, a successful solution might be reached. In [22,23,24,25], modelling was performed by constructing a stack of MEAs’ equivalent circuits: each element of the stack (the MEA, collector channel, and channels connecting the collectors of individual electrode spaces) is associated with an element that describes its operation. The electromotive force’s (EMF) source models a single MEA and the source’s voltage is related to the current passed through the cell according to a certain law. A chain of equivalent resistors describes the current flow found in the electrolyte-distributing channels. The current distribution in the equivalent circuit is found by solving a system of equations built on the basis of the balance of currents in the nodes, as well as voltage drops and EMF in the internal circuits according to Kirchhoff’s laws.

To solve this system, it is necessary to set the parameters included in it, namely, the resistances of the MEAs, supply channels, and collector channels, whose values cannot be measured directly in a traditionally designed stack. As a result, the application of this type of model to a real stack is based on solving an inverse problem, i.e., finding such values for collector resistances and channel resistances when the predictions of the model are in the best agreement with the results of the model experiment [18,19,20].

In this work, a new design for a VRFB stack is proposed. The stack design uses openable bipolar plates, consisting of two sheets of carbon material separated by an insulating gasket, wherein each carbon sheet corresponds to a metal contact exposed to the outside. Closing these contacts makes them equivalent to a traditional bipolar plate. Opening these contacts makes it possible to measure the resistance of the electrolyte supply channels for each of the electrode spaces. To measure the resistance of the electrolyte supply channels, a procedure by means of impedance spectroscopy is proposed.

The model for calculating the distribution of currents in a stack, proposed in [25,26,27], makes it possible to calculate the value of SC based on the values of equivalent resistances from which the stack circuit is built. Due to the special design of the VRFB stack, the equivalent resistances can be substituted by experimentally measured values. A comparison of the calculated and experimental dependences of coulombic efficiency (*η*_C_) on the value of the passed current makes it possible to distinguish the contribution of SC to the overall drop in charge efficiency and determine the “self-discharge” factor—the crossover of vanadium compounds.

## 2. Materials and Methods

### 2.1. Current Distribution Calculation in the VRFB Stack

**Figure 1:**
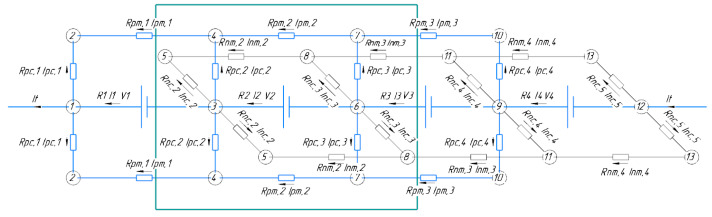
Equivalent circuit of a battery composed of 4 MEAs (N = 4). In this Figure, MEAs are given for brevity, the real number is N = 10, and the description of each additional internal MEA can be added by translation of internal fragments of the scheme. Repeating internal element is highlighted by green frame.

To calculate current distribution in electrically conductive components of stack of N MEAs, the equivalent circuit (see Figure 1) that was used and tested in similar calculations by the authors of [22,24] applied for kilowatt-scale stacks was taken as a basis.

The representation of the MEA battery in the form of an equivalent circuit (Figure 1) implies that the description is given for stationary modes of its operation; the only variable indicator is the change in state of charge (SOC) of the electrolyte during the current flow. The latter occurs evenly over the entire volume of the electrolyte. It is also assumed that the polarization characteristic of each MEA is linear (characterized by the *R*_MEA_ value), which, like the values of all other accepted resistors in the circuit of Figure 1, do not change with the composition of the electrolyte during charging/discharging.

Model development is based on the typical stack design, where the in series electrical connection of the MEA (designated “EMF force” in the diagram) is implemented using bipolar plates that hydraulically separate the oppositely charged electrodes of neighboring elements but ensure their electrical contact with zero resistances. The main (collector) electrolyte supply channels (two per posolyte and negolyte) are located along all MEAs. Each of these channels are divided into N−1 sections, according to the number of gaps between the MEA, and each such section in equivalent circuit corresponds to resistance *R*_pm_ (positive manifold) or *R*_nm_ (negative manifold). The currents flowing in these sections are designated *I*_pm,i_ and *I*_nm,i,,_ respectively. The outputs of small channels are connected to the collector channel, providing electrolyte supply/removal from electrode space of each electrode to the corresponding collector. In equivalent circuit, each such channel corresponds to a resistor with resistance *R*_nc,i_ and *R*_pc,i_ as well as currents *I*_nc,i_ and *I*_pc,i_ (negative and positive collector channels, respectively).

Each MEA in equivalent circuit generates some EMF value (*V*_0_) depending on the current value of the battery’s SOC, as follows:

*V*_0_ = *V*_cell_^0^ + 2*RT*/*F* ln SOC/(1 − SOC) (1)

Equation (1) is obtained from the Nernst equations for positive and negative electrodes—both of those initially contain equal concentrations of the oxidized and reduced forms of corresponding electroactive component in posolyte ([*Ox*^0^*_p_*] = [*Red*^0^*_p_*]) or in negolyte ([*Ox*^0^*_n_*] = [*Red*^0^*_n_*])—as well as from the determination of SOC value [0 < SOC < 1]:

SOC = [*Ox_p_*]/[*Ox_p_*^0^] = *Red_n_*/*Red*^0^*_n_*. (2)

where [*Ox_p_*] и [*Red_n_*] are current concentration values.

With current *I*_t_ (terminal current) flowing through terminals of the battery, voltage value *V*_i_ of each MEA differs from *V*_0_ in the polarization value determined by the current *I*_i_ flowing through the MEA multiplied by the internal resistance of MEA *R*_MEA_ (not indicated in the diagram, Figure 1):

*V*_i_ = *V*_0_ − *I*_t_
*R*_MEA_ (3)

Current distribution in this equivalent circuit is described by a system of equations that determine the balance of currents entering and leaving each of the circuit nodes as well as balance of EMF sources and voltage drops in resistors forming circuits based on Kirchhoff’s laws. It is necessary to find N values *I*_i_, 2N values of the currents in electrode channels for posolyte and negolyte (*I*_pc,I_ and *I*_nc,i_), and 2(N−1) values of currents in N−1 sections of collector channels (*I*_pm,i_ and *I*_nm,i_). In both cases, a multiplier of 2 is used instead of 4 because the identity of the inlet and outlet channels, both the electrode and collector, is implied; the “upper” branch of the shunt circuits’ posolyte in Figure 1 is equivalent to the “lower”, while the “near” branch of the negolyte’s distribution channels is equivalent to the “far” branch. Thus, determination of current distribution in a battery of N MEAs during current flow through terminals requires the solution of 5N−2 equations for 5N−2 currents listed above:

- along the central axis:

(4) $$I_{k\;}-I_{k-1}=2I_{\mathrm{pc},k}+2I_{\mathrm{nc},k}\;\mathrm{for}\;1\;\leq \;k\;\leq \;N+1,$$

where

(5) $$I_{0\;}=I_{N+1}=I_{t}$$

(6) $$I_{\mathrm{nc},1}=I_{\mathrm{pc},\;N+1}=0$$

- along each pm axis:

(7) $$I_{\mathrm{pc},k\;}=I_{\mathrm{pm},k-1}-I_{\mathrm{pm},k}=0\;\mathrm{for}\;1\;\leq \;k\;\leq \;N,\;$$

where

(8) $$I_{\mathrm{pm},0\;}=I_{\mathrm{pm},N}=0$$

- along each nm axis:

(9) $$I_{\mathrm{nc},k}=I_{\mathrm{nm},k-1}-I_{\mathrm{nm},k}=0\;\mathrm{for}\;2\;\leq \;k\;\leq \;N+1,$$

where

(10) $$I_{\mathrm{nm},1\;}=I_{\mathrm{nm},N+1}=0$$

- along a closed cycle around each MEA:

(11) $$V_{k}=R_{\mathrm{pc}}\;\left(I_{\mathrm{pc},k}-I_{\mathrm{pc},k-1}\right)+R_{\mathrm{pm}}\;I_{\mathrm{pm},k}\;\mathrm{for}\;1\;\leq \;k\;\leq \;N-1$$

(12) $$V_{k}=R_{\mathrm{nc}}\;\left(I_{\mathrm{nc},k}-I_{\mathrm{nc},k-1}\right)+R_{\mathrm{nm}}\;I_{\mathrm{nm},k}\;\mathrm{for}\;2\;\leq \;k\;\leq \;N$$

It should be kept in mind that Equations (4)–(9) are not linearly independent since summation of Equation (4) from 1 to N + 1 yields 0 after considering Equations (7) and (9). This is why one can substitute one of these equations, e.g., Equation (4), for k = N + 1.

Set of Equations (4)–(12) can be solved with respect to the unknown quantities: *I*_k_, *I*_pc,k_, *I*_nc,k_, *I*_pm,k_, and *I*_nc,k_. Then, one can use Equations (1) and (3) to calculate *V*_i_ for given values of *R*_MEA_ and SOC. Therefore, the resulting current distribution within the framework of this model turns out to depend on the following parameters: *R*_MEA_, SOC, *R*_pc_, *R*_nc_, *R*_pm_, *R*_nm_, and N. This calculation was performed according to an iterative algorithm, where for a given SOC according to Equations (1) and (3) the initial approximations of *V*_i_ were calculated under the assumption that *I*_i_ = *I*_t_, after which the solution of Equation (6) gave the first approximation of values *I*_i_, *I*_pc,i_, *I*_nc,i_, *I*_pm,i_, and *I*_nc,i_. The determined values of *I*_i_ were substituted into Equation (3), giving the next approximation for *V*_i_, which, in turn, were used to refine current distribution by solving the system of Equations (4)–(12). This process was repeated until current values did not change within the specified error at the next iterative step. Stationary current distribution obtained in this way for a series of SOC values from the interval (0,1) at positive (battery-charging process) and negative (discharging process) values of *I*_t_ allows us to estimate coulombic losses of galvanostatic charge–discharge cycle caused by existence of shunt currents in electrolyte supply system of channels using the following equation:

(13) $$\eta_{C}^{shunt}=\frac{\frac{1}{N}\sum_{i=1}^{N}I_{i}^{ch}t^{ch}}{I_{t}^{ch}t^{ch}}\cdot \frac{I_{t}^{dis}t^{dis}}{\frac{1}{N}\sum_{i=1}^{N}I_{i}^{dis}t^{dis}}$$

Here, $I_{i}^{ch}$ and $I_{i}^{dis}$ are average values of currents on *i*-th MEA of the battery for charging and discharging times ($t^{ch}$ and $t^{dis}$). The first factor determines the amount of charge transferred to electrolyte in the total charge passed through the battery at the charging stage. The second factor determines the amount of charge that enters the external circuit during discharge. Multiplication of these two factors is numerically equal to Coulombic efficiency of charge–discharge cycle considering the self-discharge of the battery by shunt currents. The more it differs from unity, the more noticeable is the deviation of *I*_i_ in MEA from the supplied/received *I*_t_ at battery terminals due to the presence of internal shunt circuits.

### 2.2. Arrangement and Fabrication of the VRFB Stack

To verify current distribution model, laboratory-scale VRFB stack was manufactured. The following materials were used to manufacture the VRFB stack: titanium sheets for end plates, Viton^TM^ sheets for sealing gaskets and rings, Teflon sheets for flow field frames and electrode gaskets, and graphite foil and copper foil for current collector plates. Before assembly, graphite foil sheets were treated by fluoropolymer solution (Ftoroplast-42, HaloPolymer, Moscow, Russia) to decrease current collector’s porosity and increase chemical stability [28]. Paraffin P-2 (Lukoil, Moscow, Russia) may also be used for this material’s modification [29]. GP-IEM 103 (Liaoning Grepalofu NewEnergy Co., Ltd., Panjin, China) membrane was used as separator (see Supplementary Materials (Figure S1)), carbon paper (SGL39AA, SGL GmbH, Germany) as electrodes, and fittings and tubing were created from PVDF plastic. An original approach for stack design was implemented. This approach consisted of creating a special design of a single MEA, which can be scaled up to the level of small laboratory stack as well as much larger scale.

An original approach for stack design was implemented. This approach consisted of creating a special design of a single MEA, which can be scaled up to the level of small laboratory stack as well as much larger scale.

**Figure 2:**
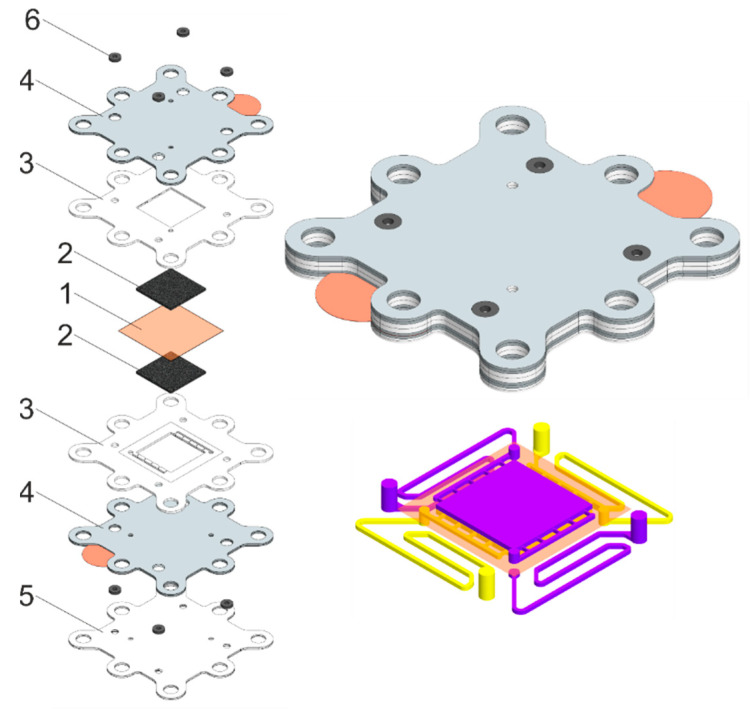
Representation of disassembled (left) and assembled (top right) repeating unit of the proposed RFB stack and scheme of electrolyte distribution channels (bottom right), where 1—ion-selective membrane, 2—electrode layers, 3—electrode gaskets, 4—current collectors, 5—flow field frame, and 6—rubber sealing of electrolyte channels. Electrolyte channel is 7.5 cm long. Flow channels in Teflon sheets for negolyte and posolyte are highlighted in purple and yellow, respectively.

The concept is as follows. The set of components for manufacturing single RFB cell or stack consists of two parts: end elements and repeating unit. Both parts include identical components, but the sequence of elements inside the assembly differs. Stack design is described in detail in Supplementary Materials (see Figures S2–S5). Components are shown in Figure 2. The VRFB stack consists of 10 single MEAs. The active area of a single MEA is 4 cm^2^.

Bipolar plates consisted of two sheets of carbon material separated by an insulating gasket, with each carbon sheet having a metal contact exposed to the outside. They act as traditional bipolar plates when the contacts are closed. If the contacts are open, the resistance of the electrolyte supply channels for each of the electrode spaces can be measured.

Electrodes with a low volumetric compression ratio were used to simplify the assurance of the uniformity of mechanical force along the stack axis at the final stage of assembly in the laboratory.

### 2.3. Determination of Charge–Discharge Characteristics of VRFB Stack

The experimental setup consists of the following parts: VRFB stack, two reservoirs with electrolytes, and tubing connecting each half-cell of MEA with corresponding reservoirs and pumps. During the tests, the electrolyte reservoirs were in an inert atmosphere.

Vanadium electrolyte was prepared from trihydrate vanadyl sulfate VOSO_4_*3H_2_O (Reakhim, Staraya Kupavna, Russia). Experimental studies were performed on electrolyte with a composition of 1 M V in 4 M H_2_SO_4_ (Sigmatek, Khimki, Russia). Preparation of 1 M V[+3.5] in 4 M H_2_SO_4_ was performed electrochemically by complete electrolysis of initial solutions with 1:1 volume ratio, replacement of posolyte volume, and mixing negolyte volume with equal volume of initial vanadyl solution, thus obtaining V[+3.5] electrolyte. The electrolyte (50 mL of posolyte and negolyte) was supplied by membrane pumps with 300 mL min^−1^ flow rate. Cyclic charge–discharge test was performed on PI-50 Pro potentiostat (Elins, Chernogolovka, Russia) with different current densities in voltage range 8–16 V.

### 2.4. Measurement and Calculation of Channel Resistance

**Figure 3:**
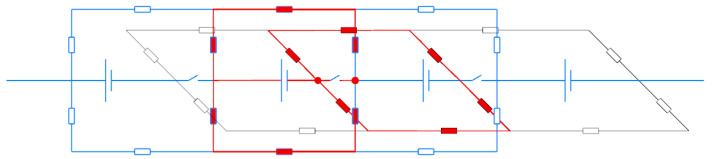
Fragment of an equivalent circuit of a stack with open-circuit graphite foil plates (highlighted in red).

Figure 3 shows a 4-MEA fragment of the equivalent circuit of the designed stack (details in Section 2.2). Instead of bipolar plates, an electrically open-circuit combination of two graphite foil plates was used: it is different from Figure 1 in that circuit breakers appear between MEAs on electron current path.

Their presence makes it possible to measure the total resistance of a part of the elements of an equivalent circuit when the terminals of the impedance meter are connected in series at circuit break points (shown in red circles). Between terminals, 4 circuits (horizontal and vertical) are connected in parallel, and these circuits are the same, i.e., *R*_measured_ = 0.25 *R*_series,_ where *R*_series_ is the resistance of one circuit. In each circuit, 2*R*_MEA_ + *R*_c_ + *R*_m_ + *R*_c_ are connected in series, thereby measuring the current flowing through the highlighted red circuits, and the resulting *R*_measured_ value is the sum of:

*R*_measured_ = 0.5 (*R*_MEA_ + *R*_c_) + 0.25 *R_m_* (14)

Considering that *R_MEA_* < *R_m_* << *R_c_*, we can assume with sufficient accuracy

*R*_c_ = 2*R*_measured_ (15)

Equation (15) was used to measure channel resistance in the vicinity of each of the 9 circuit-breaking (electrically separable) plates between adjacent MEAs. The latter was carried out by the method of electrochemical impedance spectroscopy in frequency range 0.1–50 kHz, where resistance was determined by high-frequency cutoff of the hodograph from the real axis. During this measurement, the electrolyte with 1 M V 4 M H_2_SO_4_ composition circulated through the stack.

For each of the 9 connection points, *R*_measured_ value was obtained, and its corresponding *R*_c_ was calculated and averaged over the entire stack *R*_c_ = 2327 ± 98 Ohm.

An alternative method for estimating *R*_c_ and *R*_m_ was based on the known value of conductivity of VRFB electrolyte with a composition of 250 mS cm^−1^ [17] and channel geometry (see Figure S2 in the Supplementary), i.e., its cross-section and length. According to this calculation, *R*_m_ = 7 Ohm and *R*_c_ = 2344 Ohm. The latter value is in a good agreement with the measured *R*_c_.

### 2.5. Measurement of MEA Equivalent Resistance

Experimental determination of MEA resistance used in the stack was carried out in a separate experiment with a single cell 2 × 2 cm^2^. Two methods were used: electrochemical impedance spectroscopy and the method of stationary voltammetry. Measurements by the EIS method were performed in the frequency range of 50 kHz–1 kHz with an amplitude of 20 mV at OCV, while an electrolyte consisting of 1 M of vanadium salts in 4 M of sulfuric acid at SOC 50 was supplied to the cell at a rate of 60 mL min^−1^. A stationary voltammogram was obtained by the method of sign-alternating galvanostatic polarization with an increasing value of the polarizing current in the range from ±2.5 mA cm^−2^ to ±1000 mA cm^−2^, with a duration of 25 s for each stage and registration of a stationary voltage. The resistance value was obtained from the slope of the stationary voltammogram. Figure S6a,b in Supplementary Materials present the results for both methods. The determined resistance values are *R*_EIS_ = 0.1 ± 0.01 Ohm and *R*_VA_ = 0.2 ± 0.03 Ohm. In further calculations, we used the resistance value MEA *R*_MEA_ obtained from the results of stationary voltammetry. Under the experimental conditions, the voltammogram had a linear form that satisfies the description accepted in the model (see Equation (3)).

## 3. Results and Discussion

### 3.1. Stack Performance

**Figure 4:**
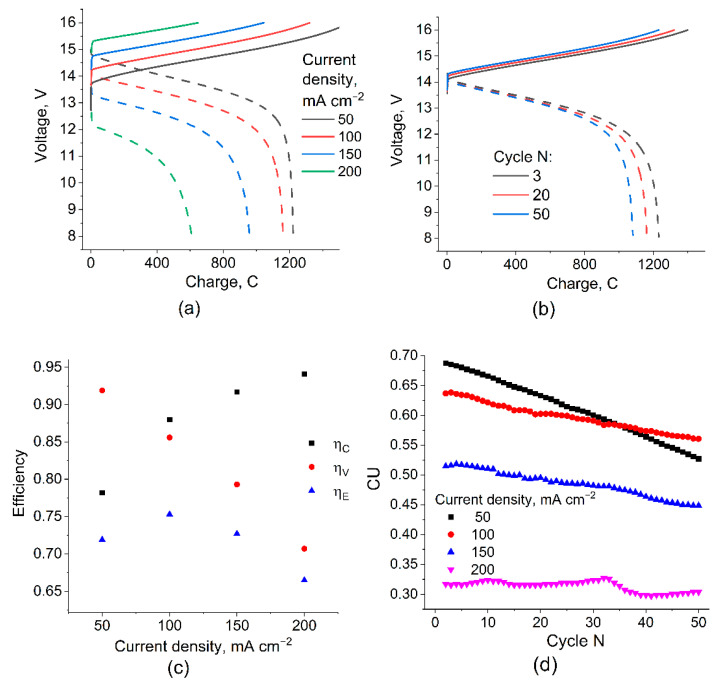
Charge–discharge curves for 10-cell VRFB with 2 × 2 cm active area at different current densities (**a**) and for different number of cycles at 100 mA cm^−2^ (**b**), as well as results of determining the cycle efficiencies (**c**) and the capacity utilization (**d**) during galvanostatic cycling test. Data (**a**,**c**) are given for 20th cycle. Full set of data obtained is presented in the Supplementary Materials (Figures S7–S18).

The measurement of the redox cycle characteristics of the manufactured stack was performed in a galvanostatic mode, with varying current density from 50 to 200 mA cm^−2^ and with a step of 50 mA cm^−2^ during 50 charge–discharge cycles for each value. Examples of the obtained voltage versus time dependences are shown in Figure 4a,b.

With an increase in the charge density, a regular voltage increase at the charging stage and a voltage drop during discharge are observed (Figure 4a, solid and dashed lines, respectively), while the amount of charge passing through the discharge unit in the voltage range of 8–16 V naturally decreases, which leads to a noticeable decrease in capacity utilization (CU). During the test, for each series of 50 cycles at a certain current density, a characteristic change in the shape of the voltage–charge curves (Figure 4b) and a gradual decrease in CU (Figure 4c) were also noted, causing the so-called electrolyte imbalance due to the change in volume, total content, and average valence of the vanadium ions in the posolyte and negolyte [30,31]. This effect is most noticeable at low current densities since the magnitude of the “imbalancing” effect of the crossover depends little on the polarizing current and is proportional to the duration of the charge–discharge cycle.

The energy efficiency of the charge–discharge cycles obtained for different current densities reaches a maximum value of 0.75 for 100 mA cm^−2^ due to two oppositely directed trends: a decrease in η_v_ and an increase in η_c_ during the transition from low to high current densities. The value of CU and its decrease with the cycle number for 100 mA cm^−2^ also represent a compromise between the decrease in CU with an increasing current density at a given voltage range and the high rate of CU decrease with a number of cycles at low current densities. It can be concluded that for the studied VRFB stack, the optimal condition for galvanostatic charge–discharge cycling takes place at 100 mA cm^−2^.

### 3.2. Modeling Results and Comparison of Calculated η_c_ with Experiment

**Figure 5:**
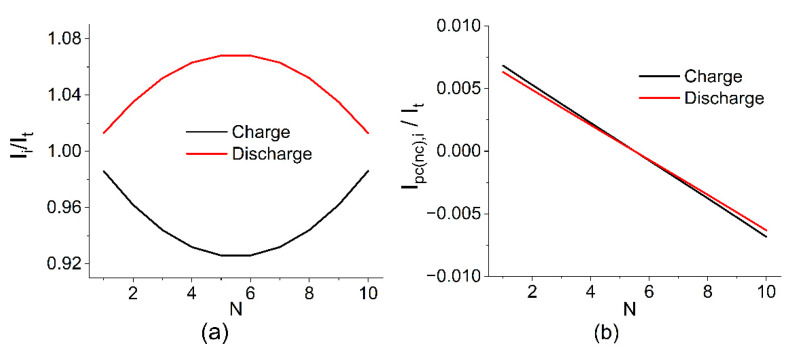

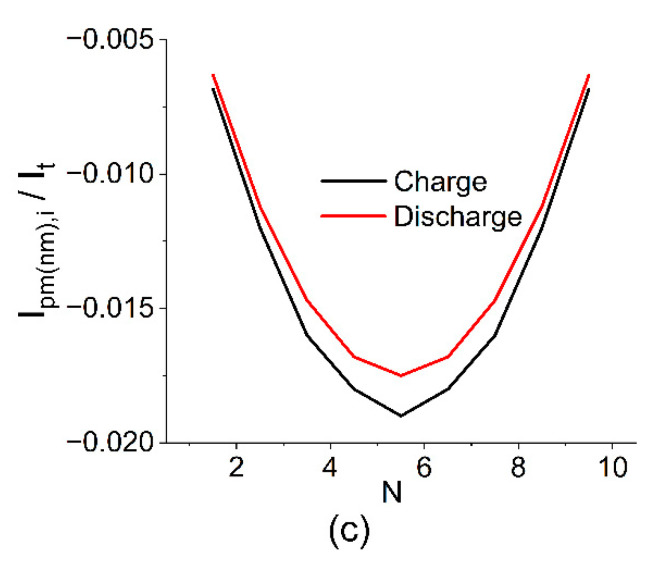
Estimated current distribution in the cell (**a**), on the electrode (**b**), and collector channels (**c**) in relation to the current during charging and discharging at SOC 50% and following the calculation parameters *V*_0_ = 1.4 V, *R*_MEA_ = 0.2 Ohm, *R*_pc_ = *R*_nc_ = 2327 Ohm, *R*_pm_ = *R*_nm_ = 7 Ohm, and N = 10.

Figure 5 shows the calculation results for the following parameters: *V*_0_ = 1.4 V, *R*_MEA_ = 0.2 Ohm, *R*_pc_ = *R*_nc_ = 2327 Ohm, *R*_pm_ = *R*_nm_ = 7 Ohm, and N = 10. This set of parameters was obtained based on experimental measurements taken on a battery of 10 MEAs with a 4 cm^2^ area for a charge–discharge current of 400 mA (100 mA cm^−2^).

The calculation results show that the highest shunt currents flow at the outermost MEAs of the battery and their influence is compensated by internal MEAs where, as a result, the maximum deviation between the local current (*I*_i_) and the one applied to the terminals (*I*_t_) is observed. The calculated value for the distribution shown in Figure 4 is 0.91, i.e., 9% of the electrolyte capacity is lost due to the presence of parasitic circuits and the self-discharge of the currents running through them.

**Figure 6:**
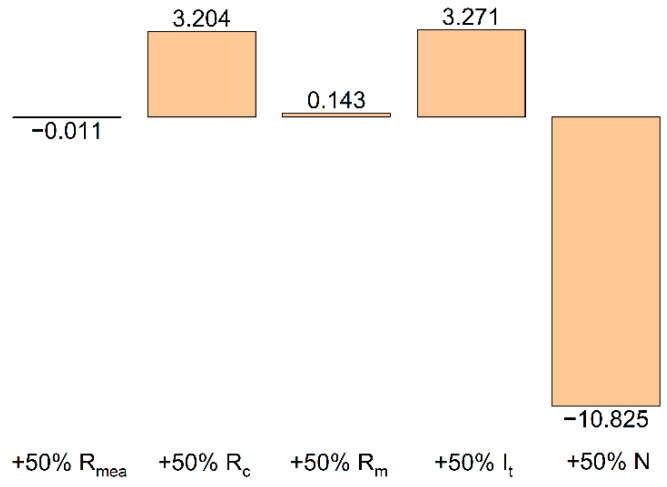
Relative increase (decrease) of shunt currents with an increase in one of the model parameters by 50% (in % relative to the value 0.91 obtained for the basic set of parameters in Figure 4).

The contributions of various quantities to the total effect of shunt currents on η_C_ have been detailed in a number of works [22,23,24,25,26,27]. According to the deterministic calculation model with accepted parameters, these contributions may be estimated from the results of a single calculation. Figure 6 illustrates the model-predicted relative effect of changing various parameters (MEA resistance (MEA resistance (*R*_MEA_), electrode channel resistance (R_c_), collector channel resistance (*R*_m_), the current applied at terminal (*I*_t_), and the number of MEAs in the battery (N)) on the value of the shunt current ($\eta_{c}^{shunt}$) with an increase in the value of the corresponding parameter by 50% relative to its base level (the values used for calculation in Figure 4). This calculation shows that for the developed battery design and selected composition of electrolyte, the coulombic efficiency of the charge–discharge cycle will be practically unaffected by a change in the resistance value of a single cell (*R*_MEA_) and the geometry of the collector channel (*R*_m_). A 50% increase in the charge/discharge current, as well as a 50% increase in the resistance of the electrode channel (for example, due to an increase in its length), will increase $\eta_{c}^{shunt}$ by 3.2 and 3.3%, respectively. On the contrary, an increase in the number of cells in the battery to 15 will lead to a decrease in the amount of extracted charge in relation to the accumulated charge by almost 11%. This circumstance should be considered when designing batteries with a large number of MEAs, alongside comparing the beneficial effect of an increase in the voltage of the discharge unit with the negative effect caused by an increase in charge losses due to shunt currents.

**Figure 7:**
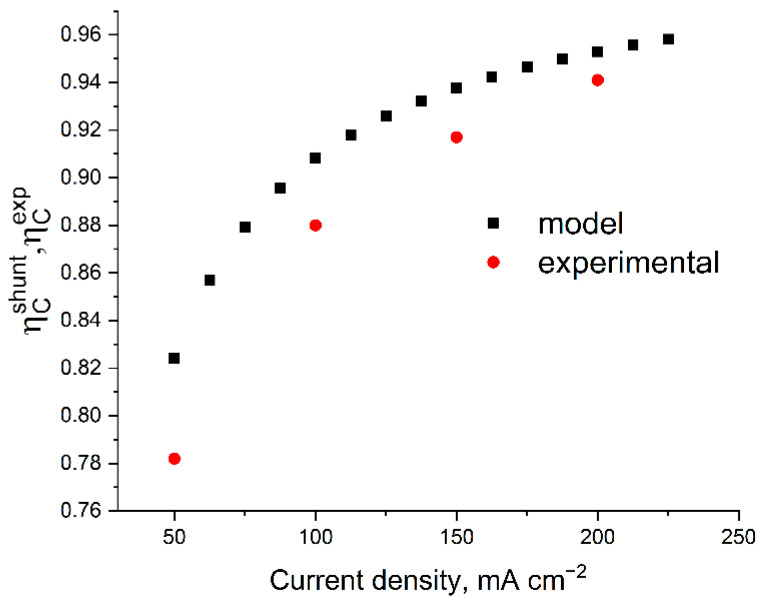
Comparison of the calculated and experimental efficiency of the galvanostatic charge–discharge cycle of a 10-cell battery with 4 cm^2^ area at different current densities. The calculation parameters correspond to Figure 4; the experimental data were measured under the following conditions: 50 mL of posolyte and negolyte with 1 M V 4 M H_2_SO_4_ composition at 300 mL min^−1^ flow rate in the voltage range of 8–16 V.

The main reasons for coulombic losses (i.e., the difference in *η*_C_ from unity) are the crossover of vanadium compounds through the membrane and shunt currents arising from the need to combine the in series electrical connection of the cells in the stack (increasing power without increasing ohmic losses) and the parallel supply of electrolytes into their electrode spaces. By comparing the result of the *η*_C_ calculation with its experimental determination, it is possible to divide the actually registered self-discharge of a battery of individual MEAs into the contributions attributed to SC (considered in the model) and the crossover of the electrolyte components through the membrane (unaccounted for in the model of the self-discharge factor). Figure 7 shows the model predictions compared with the experimentally found efficiency values of the charge–discharge cycles.

It was found that at a current density of 200 mA cm^−2^ in the voltage range of 8–16 V, the stack operates with the following parameters regarding coulombs, voltage, and energy efficiencies: *η*_C_ = 93%, *η*_V_ = 72%, and *η*_E_ = 64%. The calculation of *η*_C_ based on the experimentally measured resistances gives an overrated estimate (see Figure 6) and the difference between the experimental and theoretical *η*_C_ for each charge–discharge current density can be corrected with high accuracy by introducing a correction for voltage-independent self-discharge current by the value 2.6 mA cm^−2^, which we attribute to the effect of the crossover of vanadium compounds through the membrane. Our estimate of this value corresponds to the data of [32,33,34,35], considering the correction for the difference in the MEA composition and electrolyte concentration. Thus, by using the previously described model without crossover and electrically open-circuit bipolar plates in an experimental battery, it is possible to assess the influence of “second order” effects on the distribution of current in the stack.

## 4. Conclusions

We manufactured a 10-MEA stack with an electrically separable MEA connection, giving it the possibility to measure the resistance of transport channels, which is the main parameter of the current distribution model in the batteries of MEAs. The manufactured stack was tested in a series of galvanostatic charge–discharge cycles with the following values for these main performance indicators: *η*_C_ = 93%, *η*_V_ = 72%, and *η*_E_ = 64% at a current density of 200 mA cm^−2^ in the voltage range.

Using the proposed calculation algorithm, the current distribution in the batteries of the MEAs was calculated, and the degree of influence of these quantities (MEA resistance (*R*_MEA_), electrode channel resistance (*R*_c_), the collector channel resistance (*R*_m_), the current applied at the terminals of the battery (*I*_t_), and the number of MEAs in the battery (N)) on the coulomb efficiency of the charge–discharge cycle was estimated. It has been shown that, in addition to the number of individual cells in the MEA, *η*_C_ is also significantly affected by the magnitude of the polarizing current and the resistance of the transport channel, whereas it is not significantly affected by the resistance of a single cell (*R*_MEA_) and the geometry of the collector channel (*R*_m_).

The calculated and measured *η*_C_ were compared with respect to their charge–discharge current ranges, revealing a systematic “overestimation” of the calculated *η*_C_ compared to the experimental one. This effect is attributed to the unconsidered crossover effect of the posolyte and negolyte components through the membrane. The difference between the calculation and experiment can be almost quantitatively compensated by introducing a direct self-discharge current density of 2.6 mA cm^−2^, which correlates well with the overall crossover effect of vanadium redox forms through the membrane used.

The proposed stack design used in this work is suitable for calculating the current distributions in any MEA battery of a secondary power source using liquid electroactive components, including more cost-effective RFBs such as zinc iron. The design of a VRFB stack with open bipolar plates may be useful in laboratory practice in order to assess the influence of “second order” effects on the distribution of currents in the stack, for example, the effect of the SOC and the electrolyte flow rate on the channel resistance. In addition, an active battery (stack)-balancing system based on open bipolar plates and variable resistors installed in the gaps can be considered. Theoretically, such a design can increase the battery’s lifespan by leveling the voltage increase occurring at the outermost MEAs.

## Data Availability

Not applicable.

## Supplementary Materials

The following supporting information can be downloaded at: https://www.mdpi.com/article/10.3390/membranes12111167/s1, Figures S1–S18. Figure S1: Datasheet of a perfluorinated ion exchange membrane GP-IEM 103, which is an analogue of Nafion (Source http://www.lngpf.com/ accessed on 6 September 2022); Figure S2: Scheme of electrolyte channels (up) and two constituent (down) of proposed RFB flow field frame; Figure S3: Scheme of assembled graphite foil current collector (up) and its parts with engraved pattern (down); Figure S4: Scheme of two electrode gaskets with membrane housings; Figure S5: Disassembled repeating unit (at the left) of proposed RFB stack (at the right); where 1—ion-conductive membrane, 2—packs of electrode, 3—electrode gaskets, 4—current collectors, 5—flow field frame, 6—rubber sealing of electrolyte channels, 7 and 8—entry flow field gaskets, 9—electrolyte entrance sealing gaskets, 10—metal end plates with tubing connectors; Figure S6: Experimental determination of the MEA resistance by stationary voltammetry (a) and electrochemical impedance spectroscopy (b); Figure S7: Dependence of effective voltages of charge (black dots) and discharge (red dots) on the cycle number at current density 50 mA cm^−2^; Figure S8: CU from the cycle number at current density 50 mA cm^−2^; Figure S9: Efficiencies from the cycle number at a current density 50 mA cm^−2^; Figure S10: Dependence of effective voltages of charge (black dots) and discharge (red dots) on the cycle number at current density 100 mA cm^−2^; Figure S11: CU from the cycle number at current density 100 mA cm^−2^; Figure S12: Efficiencies from the cycle number at current density 100 mA cm^−2^; Figure S13: Dependence of effective voltages of charge (black dots) and discharge (red dots) on the cycle number at current of 150 mA cm^−2^; Figure S14: CU from the cycle number at current density 150 mA cm^−2^; Figure S15: Efficiencies from the cycle number at current density 150 mA cm^−2^; Figure S16: Dependence of effective voltages of charge (black dots) and discharge (red dots) on the cycle number at current density 200 mA cm^−2^; Figure S17: CU from the cycle number at current density 200 mA cm^−2^; Figure S18: Efficiencies from the cycle number at current density 200 mA cm^−2^.
