# Supplementary material for: Current Distribution in the Discharge Unit of a 10-Cell Vanadium Redox Flow Battery: Comparison of the Computational Model with Experiment
Source: Membranes (Basel). 2022 Nov 21;12(11):1167. doi: 10.3390/membranes12111167 (PMC9698378; doi:10.3390/membranes12111167)
Supplement: Supplementary file 1 [file membranes-12-01167-s001.zip › membranes-1931591-supplementary.pdf]

Current Distribution in the Discharge Unit of a 10-Cell Vanadium Redox Flow Battery: Comparison of the Computational Model with Experiment

Artem Glazkov [1,2,*], Roman Pichugov [1], Pavel Loktionov [1,3], Dmitry Konev [1,2,3], Dmitry Tolstel [2], Mikhail Petrov [1], Anatoly Antipov [1,2] and Mikhail A. Vorotyntsev [2]

[1] EMCPS Department, Mendeleev University of Chemical Technology of Russia, 125047 Moscow, Russia
[2] Frumkin Institute of Physical Chemistry and Electrochemistry, Russian Academy of Sciences, 119071 Moscow, Russia
[3] Institute of Problems of Chemical Physics, Russian Academy of Sciences, 142432 Chernogolovka, Russia
* Correspondence: arteymyshka@gmail.com

The datasheet of GP-IEM 103 membrane:

thickness	N mil (N=1-7)	1mil	0.0254mm
equivalent	1000g/eq	Acid capacity	1.0meq/g
Conductivity	0.1S/cm	density	2.0g/cm3
Tensile modulus	38MPa (isotropic)	Linear expansion rate	4% (isotropic)
service life	> 100000h	Shelf life	unlimited

Figure S1. Datasheet of a perfluorinated ion exchange membrane GP-IEM 103, which is an analogue of Nafion (Source http://www.lngpf.com/).

Fabrication and assembly of a repeating stack element

Components of repeating unit are shown in Figure 4 (in the manuscript). Metal end plates are fabricated from metal sheets (usually aluminum) using CNC milling machine. Compression fittings are inserted in the end plates in order to connect tubing. Fittings material must be chemically resistant to electrolytes used in the RFB (for example, PVDF or PEEK). The assembly procedure includes next steps. On the end plates sealing gasket from chemically resistant resin (for example, Viton) is placed. Then the Teflon entry gasket is used followed by Teflon flow field frame. After that two Teflon plates with engraved flow fields are placed (detailed scheme of flow frames is shown in Figure S2. Usually, the fabrication process of flow frames is quite difficult: complex milling, 3D printing or injection moulding are used for this purpose. On contrary, flow field frames from sheet materials (for example, Teflon) can be produced by stacking together several engraved plates (see Figure S2). For a RFB stack it is necessary to design flow fields channels long enough to reduce shunt current However, the increase of channels length leads to the increase of pressure drop, so these parameters should be balanced. Teflon sheets does not self-seal due to its relatively high hardness. Therefore, it is necessary to glue Teflon parts together using chemically resistant adhesive during RFB cell assembling process.

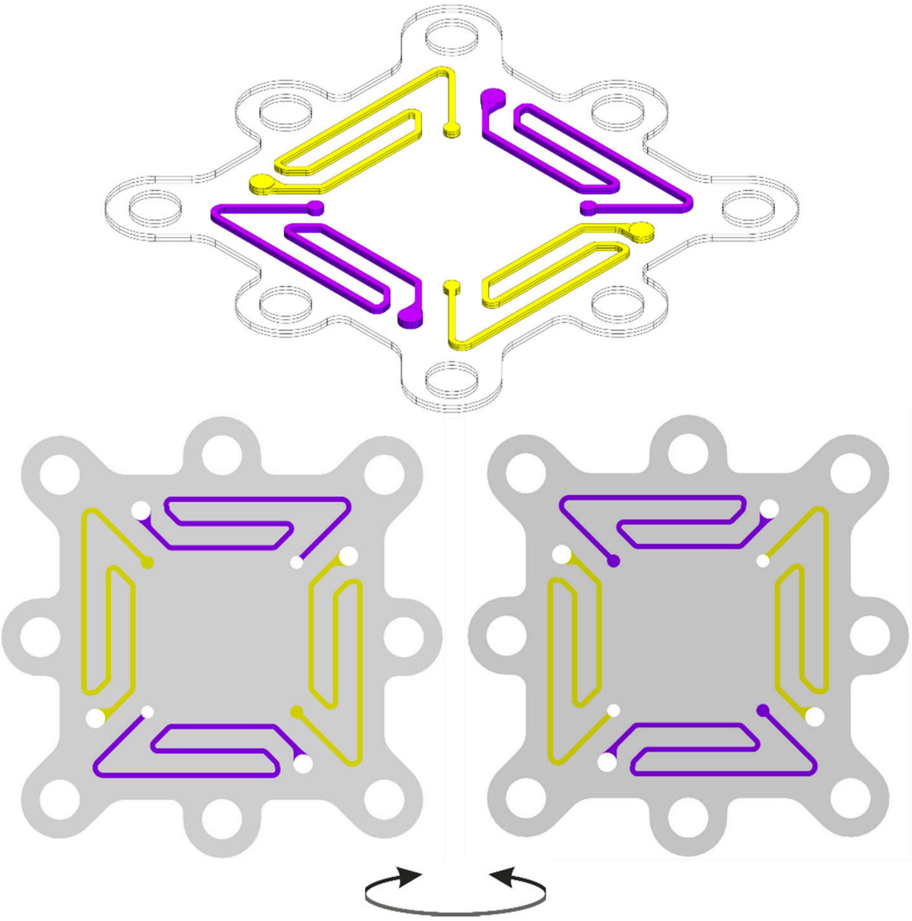

Figure S2. Scheme of electrolyte channels (up) and two constituent (down) of proposed RFB flow field frame.

After flow field frame current collector made from graphite foil and copper foil is placed (detailed scheme of the current collector shown in Figure S2). Traditionally dense graphite sheets are used for this purpose, but this material has several disadvantages: graphite is brittle, it has relatively high cost, it has high contact resistance with electrode materials and manufacturing process of bipolar plates is quite difficult. Instead, current collector plates can be created from graphite foil: sheets of flexible graphite are cheap and processable, and have a good sealing between other RFB components. Two graphite foil current collectors are made from sheet material in which orifices and profile are cut and pattern is engraved. The pattern is used to increase the impregnation degree of inert polymer (the same as mentioned above chemically resistant adhesive solution). The current collector in then assembled by pressing together two graphite foil parts with foil clamp between them.

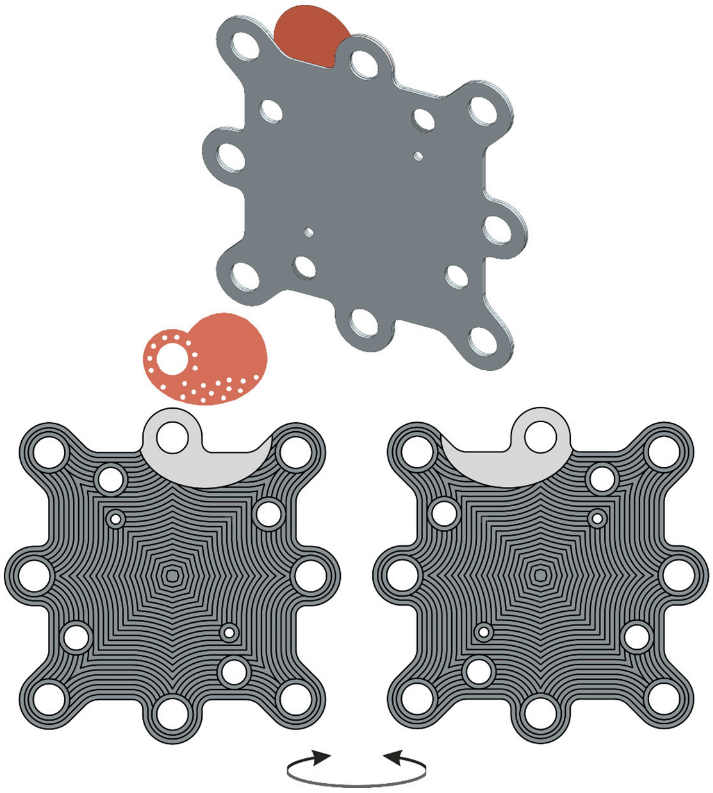

Figure S3. Scheme of assembled graphite foil current collector (up) and its parts with engraved pattern (down).

After current collector Teflon electrode gasket placed (detailed scheme described in Figure S3). There is though hole for electrode placement, channels for more uniform electrolyte distribution and engraved housing for a membrane. A membrane is sealed between two identical electrode gaskets (which are glued together) with several layers of electrode material. Benefits of such MEA are decreased shunt current due to absence of electrolyte inlet/outlet hole and decreasing of cost of battery due to smaller membrane size. For further assembling of individual RFB cell you need to insert mentioned above parts in order shown in Figure S3.

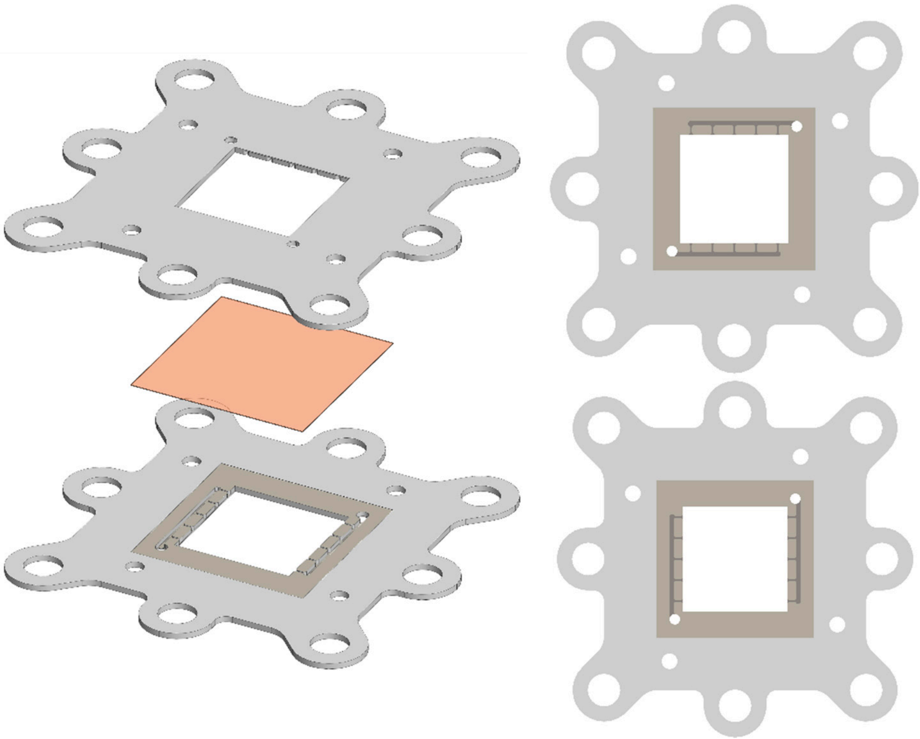

Figure S4. Scheme of two electrode gaskets with membrane housings.

Assembly of VRFB stack

To assemble RFB stack you need to introduce a series of repeating units between end elements in the shown in Figure S4 manner. The value of inserted repeating units X corresponds to the number of RFB stack MEA. Repeating unit consists of mentioned above parts: Teflon flow field, two current collector plates, two electrode gaskets with electrode layers and membrane (see Figure S4). Parallel electrolyte distribution in series of MEA implements with the use by means of cutting electrolyte inlet/outlet holes in RFB stack components.

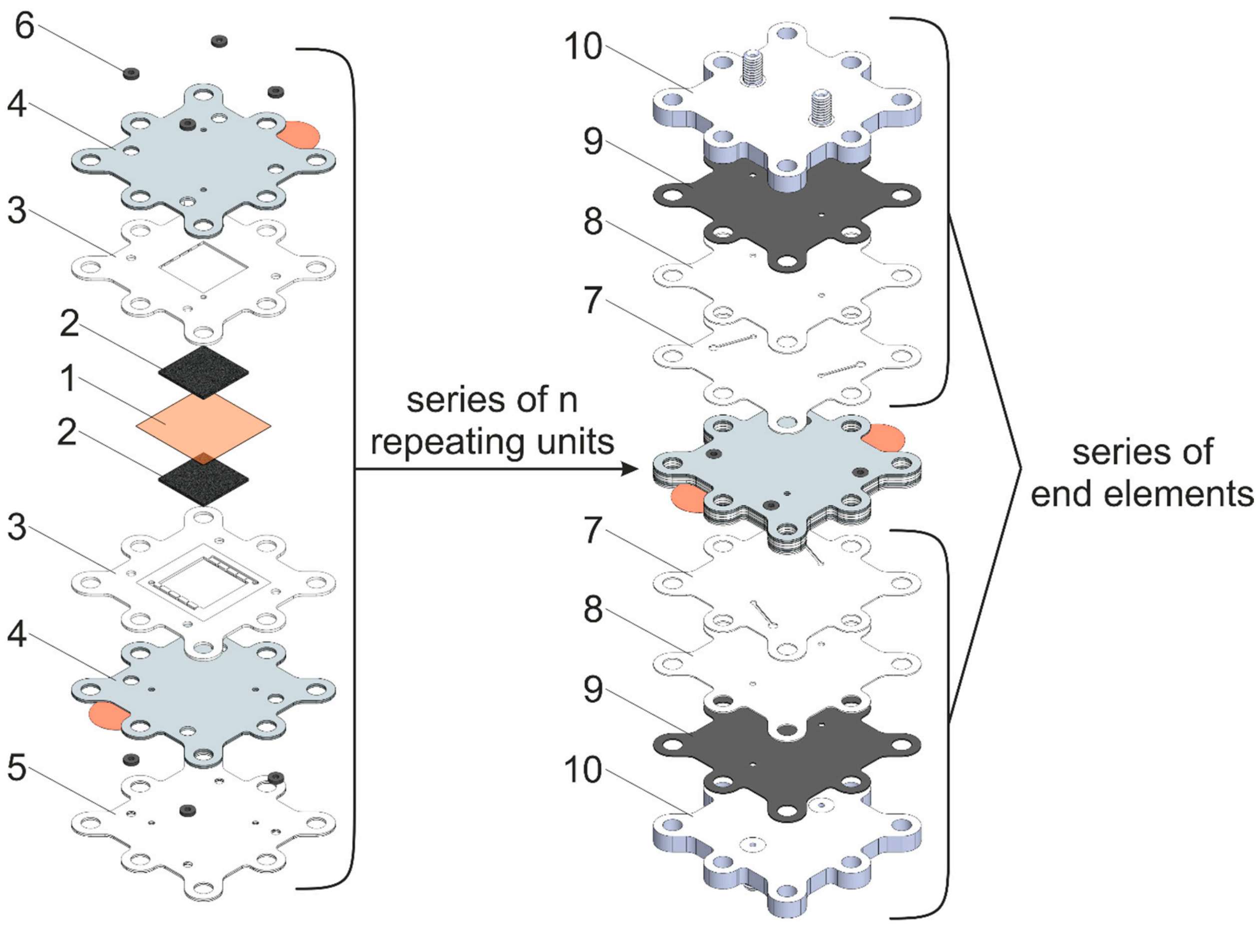

Figure S5. Disassembled repeating unit (at the left) of proposed RFB stack (at the right); where 1 – ion-conductive membrane, 2 – packs of electrode, 3 – electrode gaskets, 4 – current collectors, 5 – flow field frame, 6 – rubber sealing of electrolyte channels, 7 and 8 – entry flow field gaskets, 9 – electrolyte entrance sealing gaskets, 10 – metal end plates with tubing connectors

Those holes sealed with washers made from chemically resistant resin. It should be mentioned that in case of parallel connection of n MEAs hydraulic electrolyte velocity in each individual cell is the n-th part of overall electrolyte flow rate.

Due to relatively high flow field channel length hydrodynamic resistance of the flow frame can be significant. Cutting through holes pattern in layers of electrodes can be helpful in decreasing pressure drop in MEA half-cell and the implementation of more uniform electrolyte distribution.

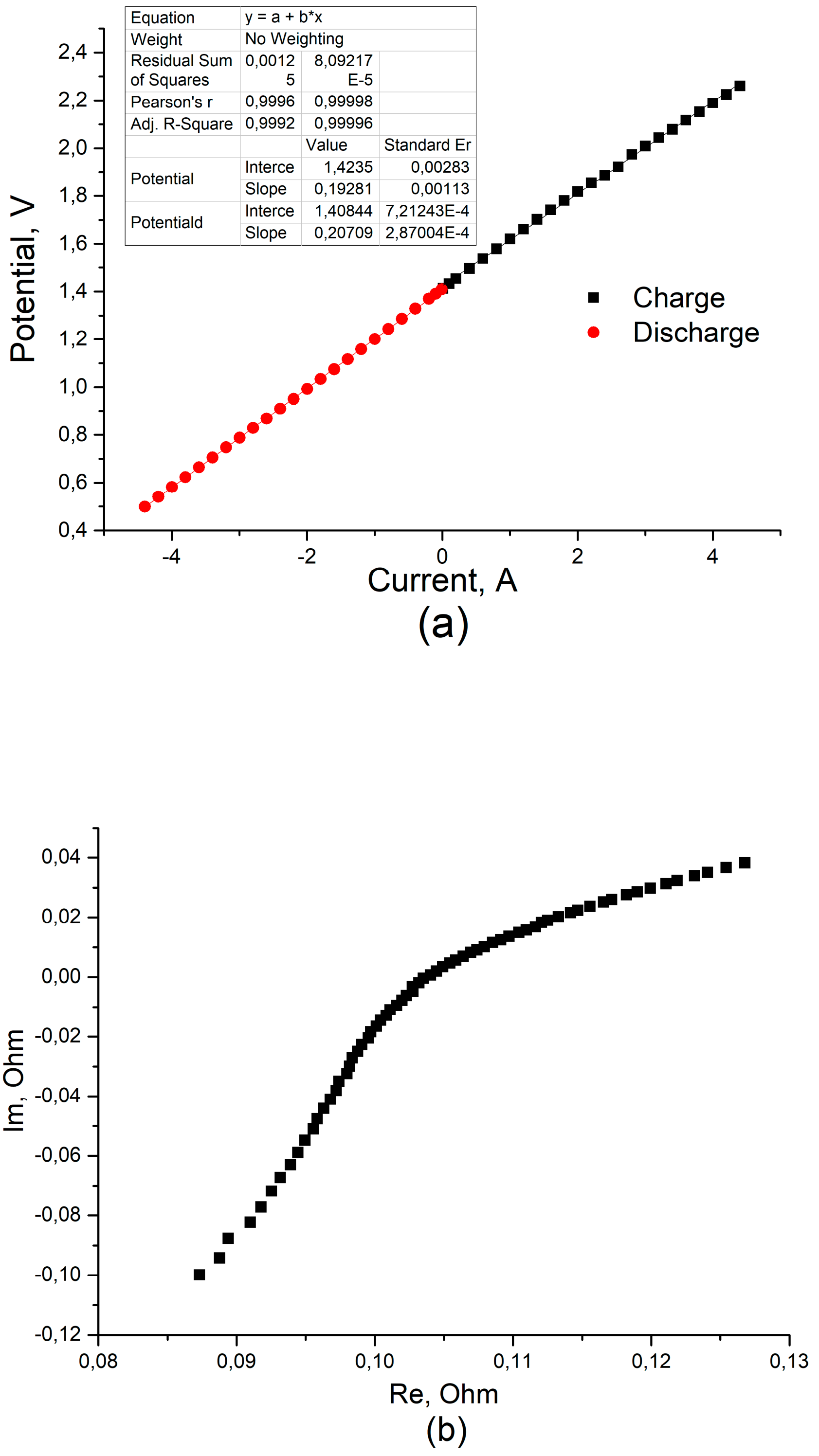

Figure S6. Experimental determination of the MEA resistance by stationary voltammetry (a) and electrochemical impedance spectroscopy (b).

Stack performance

The results of the galvanostatic charge-discharge cycle of a 10-cell battery with 4 cm^2 area at different current densities. The total initial volume of posolyte and negolyte (equal portions) was 100 mL, the composition was 1 M V 4 M H_2SO_4, composition at 300 ml min^{-1} flow rate in the voltage range 8-16 V.

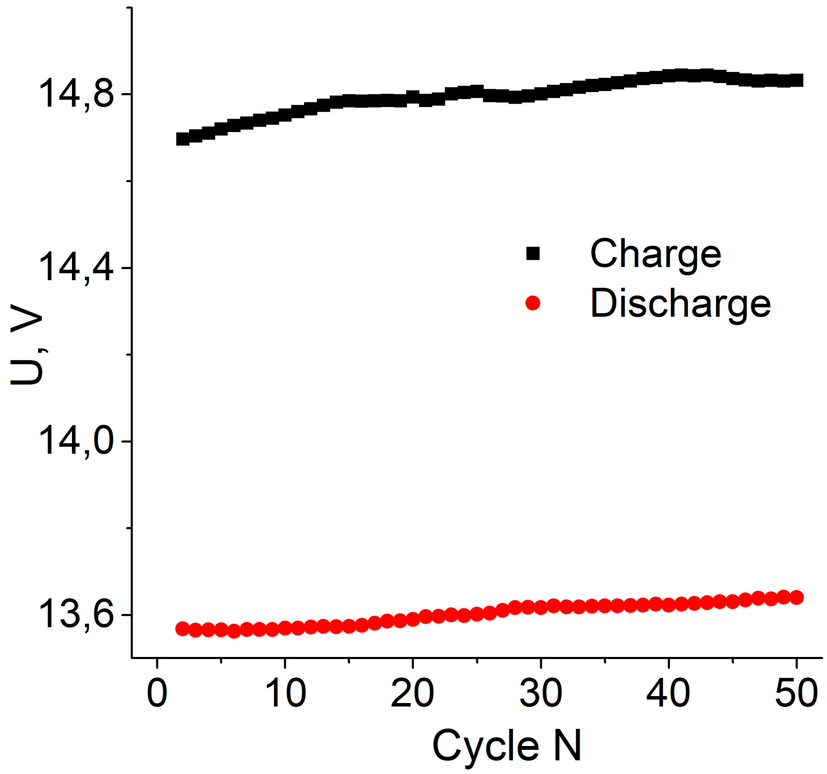

Figure S7. Dependence of effective voltages of charge (black dots) and discharge (red dots) on the cycle number at current density 50 mA cm^{-2}.

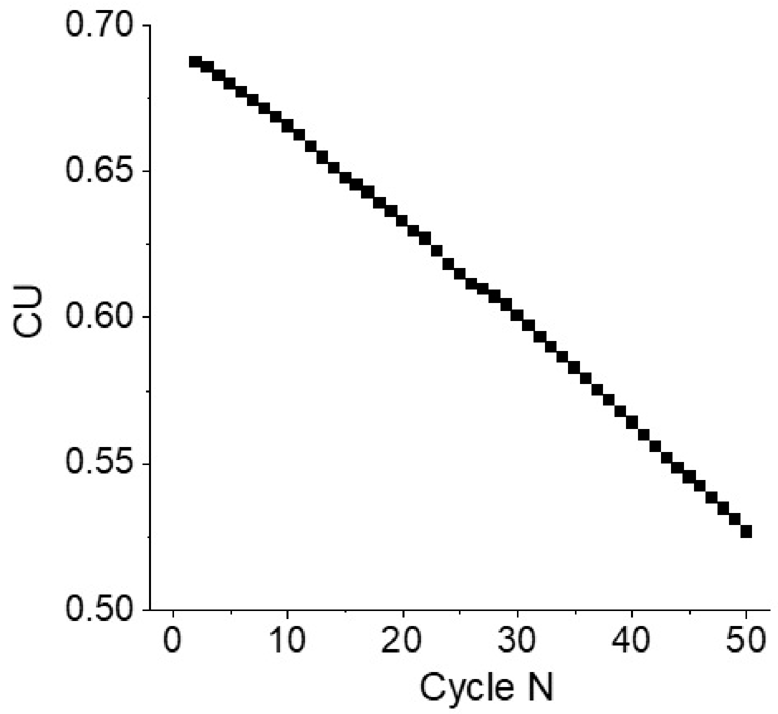

Figure S8. CU from the cycle number at current density 50 mA cm-2.

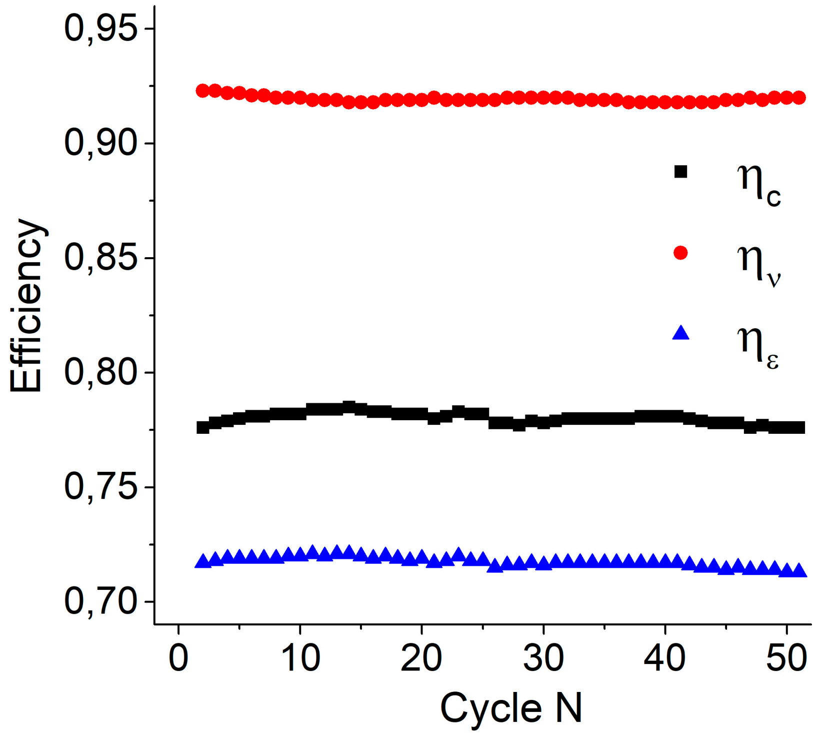

Figure S9. Efficiencies from the cycle number at a current density 50 mA cm-2.

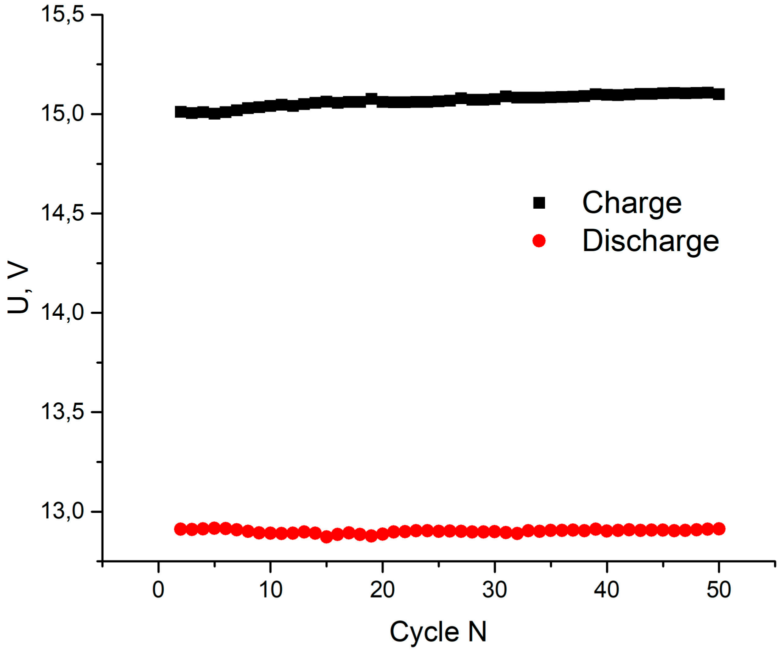

Figure S10. Dependence of effective voltages of charge (black dots) and discharge (red dots) on the cycle number at current density 100 mA cm^{-2}.

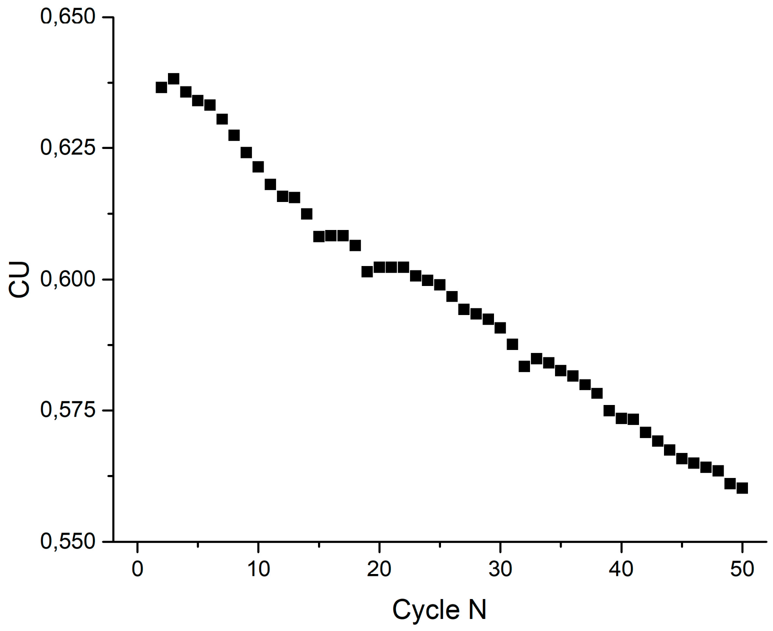

Figure S11. CU from the cycle number at current density 100 mA cm^{-2}.

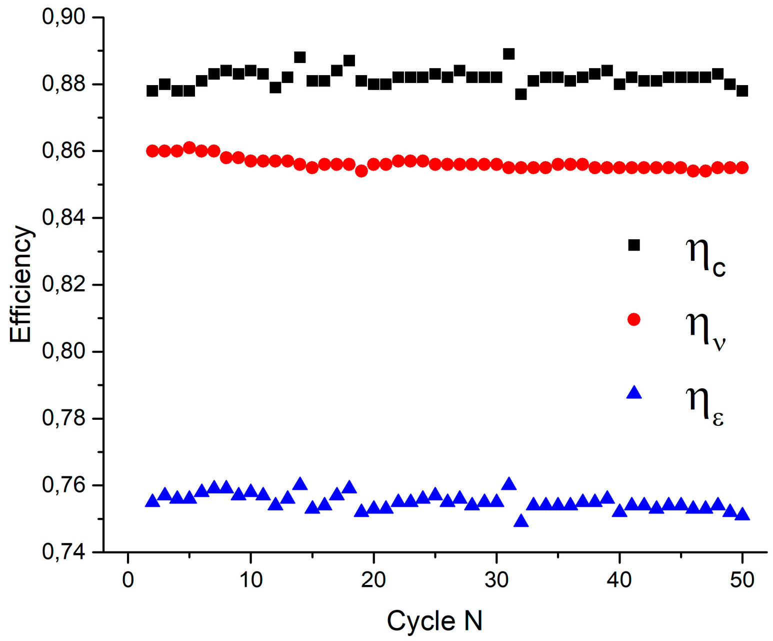

Figure S12. Efficiencies from the cycle number at current density 100 mA cm-2.

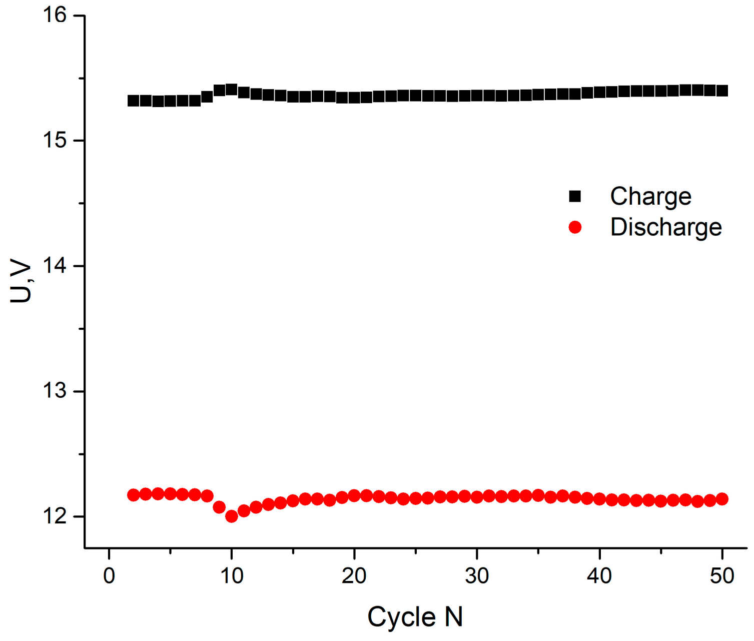

Figure S13. Dependence of effective voltages of charge (black dots) and discharge (red dots) on the cycle number at current of 150 mA cm-2.

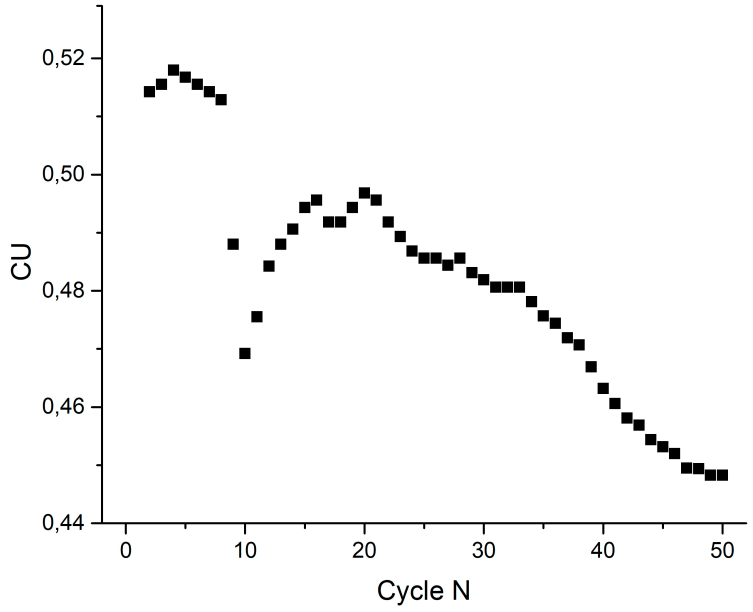

Figure S14. CU from the cycle number at current density 150 mA cm^{-2}.

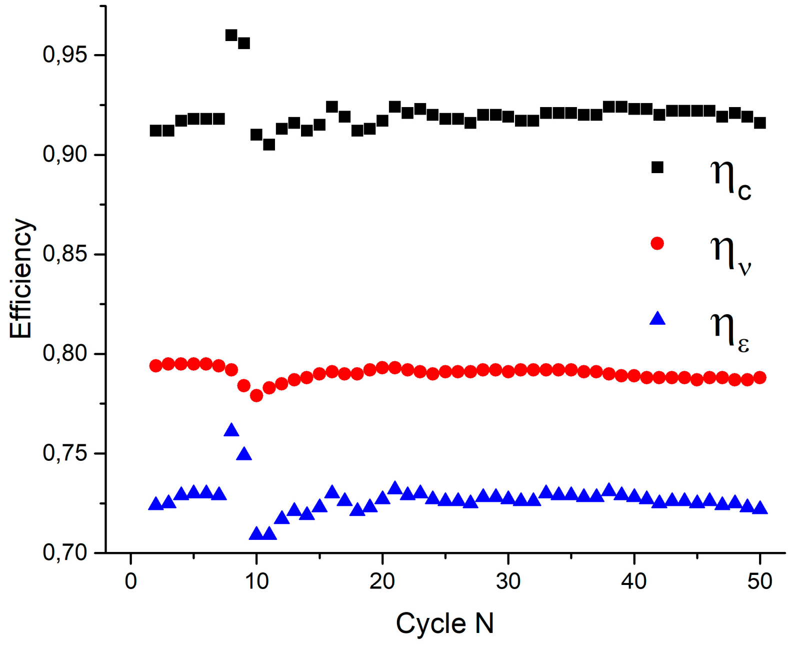

Figure S15. Efficiencies from the cycle number at current density 150 mA cm^{-2}.

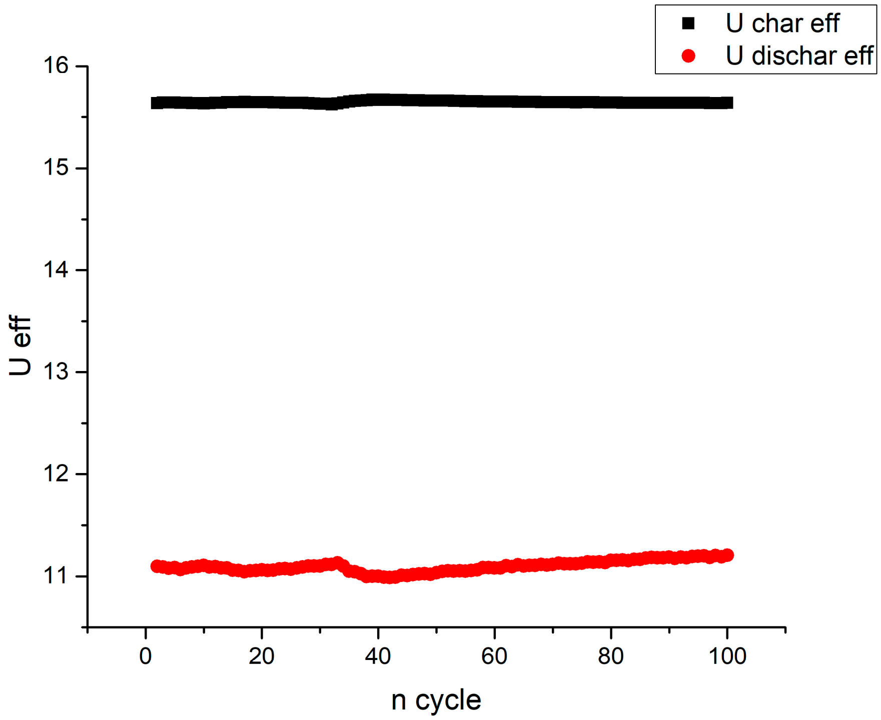

Figure S16. Dependence of effective voltages of charge (black dots) and discharge (red dots) on the cycle number at current density 200 mA cm^{-2}.

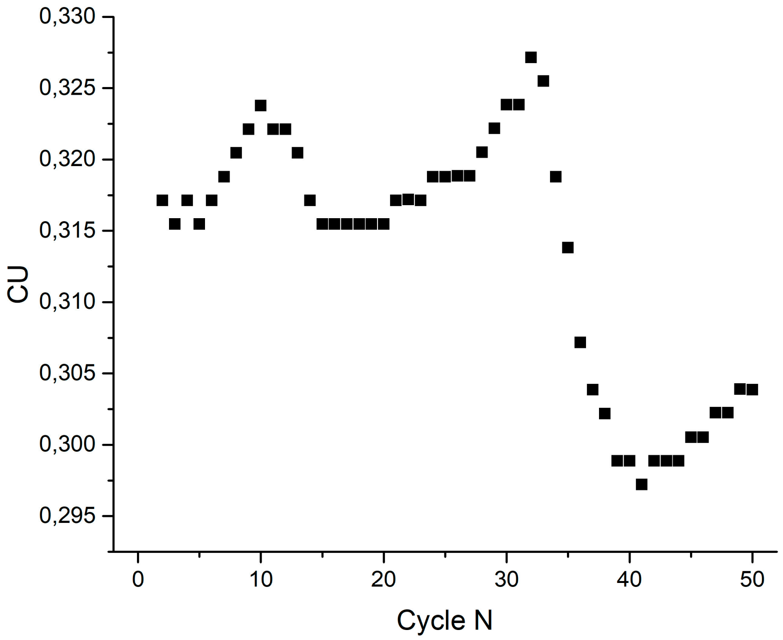

Figure S17. CU from the cycle number at current density 200 mA cm^{-2}.

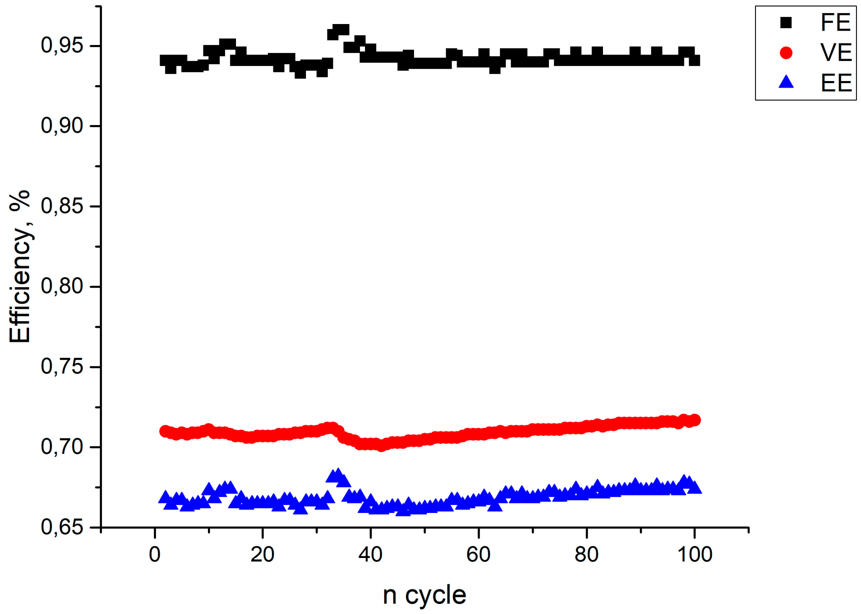

Figure S18. Efficiencies from the cycle number at current density 200 mA cm^{-2}.
